# Untargeted Metabolomics Reveals Metabolic Link Between Histone H3K27 Demethylase UTX and Neurodevelopment

**DOI:** 10.1111/jcmm.70334

**Published:** 2025-01-08

**Authors:** Lin Chen, Maozhu Liu, Xinhua Dai, Cuilin He, Kejing Wang, Jinhua Tang, Yang Yang

**Affiliations:** ^1^ Department of Pharmacy Chongqing Health Center for Women and Children, Women and Children's Hospital of Chongqing Medical University Chongqing China; ^2^ Center of Infectious Diseases, West China Hospital Sichuan University Chengdu China; ^3^ Department of Laboratory Medicine, West China Hospital Sichuan University Chengdu China; ^4^ Department of Pharmacy The First People's Hospital of Shuangliu District Chengdu China

**Keywords:** metabolic pathway, metabolomics, neurodevelopment, UTX

## Abstract

Ubiquitously transcribed tetratricopeptide repeat on chromosome X (UTX) is a chromatin modifier responsible for regulating the demethylation of histone H3 lysine 27 trimethylation (H3K27me3), which is crucial for human neurodevelopment. To date, the impact of UTX on neurodevelopment remains elusive. Therefore, this study aimed to investigate the potential molecular mechanisms underlying the effects of UTX on neurodevelopment through untargeted metabolomics based on ultra‐high‐performance liquid chromatography–tandem mass spectrometry (UPLC‐MS/MS). We found that *UTX* knockout in neurones leads to cell death and apoptosis in the hippocampus and cortex, as well as induces impaired learning and memory functions in mice. Moreover, *UTX* deletion contributed to significant metabolic perturbations in brain tissues. A total of 223 differential metabolites were identified between wild‐type (WT) and *UTX* cKO mice. Pathway analysis indicated that the metabolic pathways mainly affected by *UTX* deletion were alanine, aspartate, and glutamate metabolism, resulting in significant alterations in L‐alanine, L‐aspartate, D‐aspartate, N‐acetylaspartylglutamate, L‐glutamate, and argininosuccinic acid. These data emphasised that UTX may exert a key effect in neurodevelopment and that the underlying mechanism may be related to the regulation of the alanine, aspartate, and glutamate metabolism pathways, especially the characteristic metabolites involved in this pathway.

## 1 Introduction

Neurodevelopmental diseases, including cognitive impairment, autism, and epilepsy, are heavy health burdens in modern societies, with their prevalence still on the rise [1]. Studies have shown that neurodevelopment in early life is influenced by both genetic and environmental factors [2, 3]. The interaction of genetics and environment can cause permanent effects on brain structure and function, and some modifications may be detrimental to neurodevelopment, consequently inducing disorders such as mental health problems and behavioural dysfunctions [3]. Previous research has highlighted that some diseases, such as schizophrenia, can be prevented through interventions that target neurodevelopment during early life [4]. Therefore, it is of great importance to study the role of genes in the nervous system to gain a deeper understanding of the pathophysiological mechanisms of neurological disorders and find reliable therapeutic approaches.

Ubiquitously transcribed tetratricopeptide repeat on chromosome X (UTX, encoded by *KDM6A*), a chromatin modifier responsible for regulating the demethylation of histone H3 lysine 27 trimethylation (H3K27me3), which participates in various pathophysiological processes [5]. A growing number of studies have focused on the functional role of UTX in the nervous system, finding that it is involved in the regulation of neural differentiation, dendritic morphology, and neural stem cell proliferation and differentiation [6, 7]. The existing data indicated that 53BP1‐UTX interaction facilitates the activation of neurogenic genes that are essential for human neurodevelopment [5]. In addition, Zhao et al. reported that reduced UTX expression could induce female‐specific H3K27me3 overexpression and Mash1 transcription repression, which promotes abnormal proliferation, differentiation, and apoptosis, ultimately leading to neural developmental disorders [8]. Moreover, other research supported that *KDM6A* (*UTX*) may be the causative gene of Kabuki syndrome, an inherited disorder characterised by developmental delay and intellectual disability [9]. Taken together, the above studies imply that *UTX* deficiency may cause neurodevelopmental deficits. Despite the available evidence demonstrating a possible link between UTX and neurodevelopment, current research is still limited, and how UTX controls the neurodevelopmental processes remains largely unknown, so further studies are urgently needed to provide reliable evidence.

Nowadays, with more and more credible evidence pointing out that metabolites are closely associated with human health and disease, the systems‐level effects of metabolites deserve increasing attention [10, 11]. Metabolomics has proven to be a reliable high‐throughput strategy for characterising biospecimens, such as biofluids, cells, and tissues, with a focus on uncovering metabolic alterations caused by genetic or environmental factors [12]. Due to the complexity of endogenous and exogenous metabolites, untargeted metabolomics is routinely adopted first to identify and quantify as many metabolites as possible, thus laying the foundation for subsequent targeting studies [13]. Various analytical platforms, including liquid chromatography–tandem mass spectrometry (LC–MS) and gas chromatography‐tandem mass spectrometry (GC–MS), enable the identification of a wide range of metabolite classes and have been widely implemented in the metabolomics field [14]. Among them, ultra‐high‐performance liquid chromatography–tandem mass spectrometry (UPLC‐MS/MS) is considered one of the core analytical techniques owing to its wide selectivity and high sensitivity, which can provide comprehensive metabolic information [15].

Metabolomics is now widely used to establish links between metabolites and pathophysiological processes in the nervous system [16, 17, 18]. Numerous studies have illustrated the involvement of various metabolites in modulating neuronal development. For example, Moreau et al. conducted targeted metabolomics and found that lipids (e.g., phosphatidylcholines) are important biomarkers for childhood growth and neurocognitive outcomes [19]. Another untargeted metabolomics study showed that disruptions in multiple neurodevelopment‐related metabolic pathways, such as glycosphingolipid biosynthesis and metabolism, N‐glycan and pyrimidine metabolism, bile acid pathways, and C21‐steroid hormone biosynthesis and metabolism, may be relevant to the development of autism [20]. Together, these studies provide some evidence that metabolite alterations may be a key step contributing to neurodevelopmental abnormalities. However, to the best of our knowledge, there are no published studies investigating whether *UTX* deficiency contributes to metabolite disruptions that are associated with adverse neurodevelopmental consequences.

Herein, to broaden our understanding of the effects of UTX on the brain, we knocked out *UTX* in mouse neurones to determine the relationship between UTX and neurodevelopment. Furthermore, whole brain tissues from wild‐type (WT) and *UTX* neuronal conditional knockout (cKO) mice were collected to perform untargeted metabolomics using UPLC‐MS/MS. We hope this study can provide new insights into the potential mechanisms underlying the UTX function in neurodevelopment and offer empirical evidence and a theoretical basis for in‐depth research in this field.

## 2 Materials and Methods

### 2.1 Reagents and Solutions

High‐performance liquid chromatography (HPLC)‐grade acetonitrile, methanol, and ammonia were purchased from Thermo Fisher Scientific (Waltham, MA, USA), and ammonium acetate was purchased from Sigma‐Aldrich (St. Louis, MO, USA). Ultra‐pure water used in this study was purified using the Milli‐Q Academic System (Millipore, MA, USA).

### 2.2 Animals and Groups

*UTX* ^*f/f*^ and *Emx1‐Cre* transgenic mice were obtained from CyagenBiosciences Company (Guangzhou, China). The *UTX* cKO mice were generated by crossing *UTX* ^*f/f*^ mice and *Emx1‐Cre* transgenic mice. Neurone‐specific Emx1 (empty spiracles homologue 1) is an important transcription factor in the cerebral cortex and hippocampal development, and *Emx1‐Cre* mice have been widely used in studies of neurodevelopment [21, 22]. *UTX* ^*f/f*^ mice possess loxP sites on either side of exon 3 of the *UTX* gene. When these *UTX* ^*f/f*^ mice are mated with *Emx1‐Cre* mice, the resulting offspring will have exon 3 deleted in Cre‐expressing tissues. After birth, RNA was extracted by tail clipping, and *UTX* cKO mice were identified by PCR screen. Finally, 8–12‐week‐old male *UTX* cKO mice and age‐matched WT littermates were used for the present study. Animals were housed in a controlled environment with regulated temperature, humidity, and a 12/12 h light–dark cycle. They had ad libitum access to food and water. A five‐day adaptation period was provided for all mice before commencing the experiments. Animal experiments were conducted strictly in compliance with the Guide for the Care and Use of Laboratory Animals, and experimental procedures were approved by Chongqing Health for Women and Children Application for Laboratory Animal Welfare and Ethical Review (No. 2021001).

### 2.3 Morris Water Maze

The Morris Water Maze (MWM) test used to evaluate spatial learning and memory function in mice was conducted based on previously published protocols with some modifications [23, 24]. The MWM (63034, RWD Life Science, China) is a circular tank (120 cm diameter, 50 cm height) filled with opaque water (23°C ± 2°C) up to 20 cm and divided into four equidistant quadrants: the first, second, third, and fourth quadrants, with a transparent platform 1 cm below the water surface in a fixed location (the first quadrant). In the spatial learning trials, each quadrant was marked with pictures of different colours and shapes as spatial cues. The 8–12‐week‐old male mice were allowed to swim freely until they found the underwater platform within 60 s, and if the mice failed to reach the target platform within the allotted time, they were gently guided to reach the platform and remained there for 10 s. Each trial started in a different quadrant, and each mouse received four trials per day for five consecutive days, with a 30 min rest period between two trials. Escape latency (time to find the hidden platform) was recorded to evaluate the spatial learning ability while conducting trials. The probe memory trials were conducted 1 h after the last spatial learning trial. The submerged platform was removed, and the mice were allowed to swim freely for 60 s. The number of crossings of the platform was calculated to assess spatial memory retrieval ability. A video tracking and recording system suspended above the MWM centre was employed to acquire the swimming tracks and experimental parameters. The researchers performing the MWM test and analysing the data were blind to the animal groups.

### 2.4 H&E and TUNEL Staining

8–12‐week‐old male mice were deeply anaesthetised with sodium pentobarbital (40 mg/kg) and then slowly perfused with phosphate‐buffered saline (PBS) and 4% paraformaldehyde for 15 min. Afterward, the brains were rapidly dissected and fixed in 4% paraformaldehyde for 24 h, then embedded in paraffin and serially cut into 5 μm‐thick coronal sections. Haematoxylin–eosin (H&E) staining (Servicebio, Wuhan, China) was performed to observe the histopathological damage of the brain. Terminal deoxynucleotidyl transferase dUTP nick end labelling (TUNEL) staining (Beyotime Biotech, Shanghai, China) was used to detect apoptosis of brain cells following the manufacturer's protocol, and TUNEL‐positive cells were shown in green and the nuclei were stained in blue. H&E and TUNEL‐stained sections were photographed under 400× magnification using light microscopy and fluorescence microscopy, and cell death and apoptosis were measured in mouse hippocampal CA1 regions and cortex using ImageJ software (version 1.8.0), respectively.

### 2.5 Sample Preparation for Metabolomics

Whole brain tissues were harvested from 8 to 12‐week‐old male WT (*n* = 6) and *UTX* cKO mice (*n* = 6) immediately after decapitation under anaesthesia with 40 mg/kg sodium pentobarbital (intraperitoneal). After measurement of tissue weight, brain samples were snap‐frozen in liquid nitrogen and stored at −80°C prior to UPLC‐MS/MS analysis. The sample processing procedure was based on the published protocols with some modifications [25]. Briefly, frozen brain tissues were thawed slowly at 4°C, and then 800 μL of pre‐cooled methanol/acetonitrile/water solution (2:2:1, *v*/*v*) was added to 80 mg of brain samples. After vortex‐mixing (1 min), sonication (30 min), and standing at −20°C (10 min), the mixture was centrifuged (14,000 *g*, 20 min, 4°C), and the supernatant was dried under vacuum at room temperature. Subsequently, 100 μL of acetonitrile aqueous solution (acetonitrile: water = 1:1, *v*/*v*) was added to re‐dissolve the dried samples, and after vortex‐mixing (1 min) and centrifugation (14,000 *g*, 15 min, 4°C), the supernatant was finally transferred for analysis. Meanwhile, the quality control (QC) samples were prepared by mixing equal volumes of each brain sample and analysed after every 6 original samples to monitor the stability and performance of the instrument.

### 2.6 UPLC‐MS/MS Analysis

Brain metabolomic analysis was performed using an Agilent 1290 liquid chromatography system (Agilent, USA) equipped with quadrupole time‐of‐flight (AB Sciex TripleTOF 6600). Metabolites were separated on an ACQUIY UPLC BEH Amide column (1.7 μm, 2.1 mm × 100 mm, Waters, Ireland), with the column temperature maintained at 25°C. In both positive and negative ion modes, 2 μL of supernatant was injected, and the optimal mobile phases consisting of 25 mM ammonium acetate and 25 mM ammonium hydroxide aqueous solution (phase A) and acetonitrile (phase B) were used for the elution at a flow speed of 0.5 mL/min during the analysis. The elution gradients were set as follows: 95% B, 0–0.5 min; 65% B, 0.5–6.5 min; 40% B, 6.5–8.5 min; 95% B, 8.5–8.6 min, with a final equilibration for 3 min.

MS analysis was carried out on an AB SCIEX Triple‐TOF 6600 (AB Sciex, USA) with an electrospray ionisation (ESI) source in positive and negative ion modes. The ESI parameters were set as follows: Ion Source Gas1 (Gas1) as 60, Ion Source Gas2 (Gas2) as 60, curtain gas (CUR) as 30, source temperature: 600°C, IonSpray Voltage Floating (ISVF) ± 5500 V. The *m*/*z* range of the apparatus was 60–1000 Da, and the TOF MS scan accumulation time was 0.20 s/spectra for MS‐only acquisition. For automated MS/MS acquisition, the *m*/*z* range was 25–1000 Da, and the product ion scan accumulation time was set to 0.05 s/spectra. Information‐dependent acquisition (IDA) in high sensitivity mode was selected to acquire product ion scans with the following parameters: collision energy (CE) fixed at 35 V with ±15 eV; declustering potential (DP); 60 V (+) and −60 V (−); exclude isotopes within 4 Da, and 10 candidate ions to monitor per cycle.

### 2.7 Data Processing

UPLC‐MS raw data were converted to MzXML files using ProteoWizard MSConvert and then imported into the XCMS software (XC‐MS plus, CA, USA) for peak picking and grouping. The detailed peak picking parameters were set as follows: centWave *m*/*z* = 10 ppm, peakwidth = c (10, 60), prefilter = c(10, 100). For peak grouping, bw = 5, mzwid = 0.025 and minfrac = 0.5 were used. The resultant data matrix was processed for isotope and adduct annotation using CAMERA (Collection of Algorithms of MEtabolite pRofile Annotation). Only variables lost in less than 50% of the samples could be retained. Metabolite identification was based on molecular weight‐to‐charge ratio (*m*/*z*) (< 10 ppm) and mass spectrometry, with reference to an in‐house database established using available authentic standards.

### 2.8 Statistical Analysis

To show differences in metabolite composition between groups, the processed data were transferred to the R software package for partial least squares‐discriminant analysis (PLS‐DA). The performance of the PLS‐DA model was assessed by a 200‐times permutation test. Variable influence on projection (VIP) values were generated in the PLS‐DA model to elucidate the contribution of metabolites to classification, and *p*‐values from the Student's *t*‐test were used to detect differences in metabolites between groups. Metabolites that satisfied the screening conditions of VIP > 1.0 and *p* < 0.05 were considered significantly different. Next, volcano plots and heat maps were generated to visualise the metabolite differences between different groups. We further used Pearson's correlation analysis to determine the correlation between two metabolites. Metabolic pathway analysis was carried out using MetaboAnalyst 6.0 (https://www.metaboanalyst.ca/) to obtain the most relevant pathways based on the criteria of *p* < 0.05 and impact score > 0.3.

Data were subjected to an unpaired two‐tailed Student's *t*‐test using GraphPad Prism 8 Software (GraphPad, San Diego, CA, USA) to assess statistical significance between the two groups. All data were presented as mean ± SD. *p* < 0.05 was considered a statistically significant difference.

## 3 Results

### 3.1 UTX Neuronal cKO Mice Exhibit Spatial Learning and Memory Impairments

**FIGURE 1:**
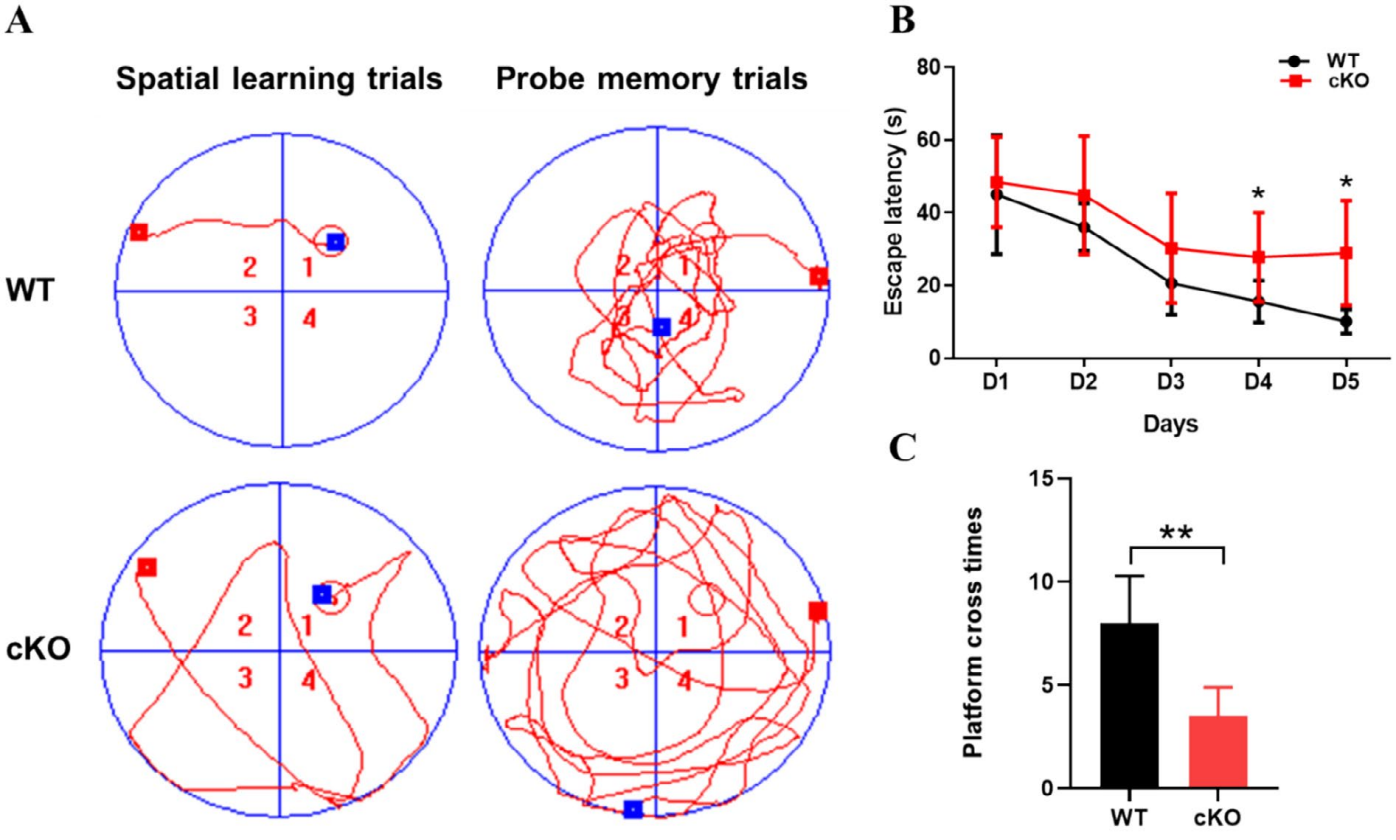
Results of the Morris Water Maze test. (A) Representative path patterns of WT and *UTX* cKO mice in spatial learning trials and probe memory trials. Empty circles in the first quadrant indicate hidden platform locations. Escape latencies (B) and platform cross times (C) were compared between the WT and *UTX* cKO groups by Student's *t*‐test. All data were expressed as mean ± SD (*n* = 6). *p < 0.05, **p < 0.01 versus WT group.

Cognitive deficits are one of the common adverse outcomes of neurodevelopmental abnormalities. First, the present study utilised the MWM test to assess hippocampal‐dependent spatial cognitive abilities. Figure 1A displays representative swimming tracks for the spatial learning trials and probe memory trials. As shown in Figure 1B, in the first day of the spatial learning trials, there was no significant difference in escape latency between the two groups. During the five training days, the escape latencies of WT and *UTX* cKO mice were progressively shorter. On the fourth and fifth days, mice in the *UTX* cKO group took significantly longer (*p* < 0.05) to find the hidden platform than mice in the WT group, implying that *UTX* deletion resulted in worse learning ability. Besides, loss of *UTX* in neurones was found to decrease the number of platform crossings (*p* < 0.01) in probe memory trials, which means *UTX* cKO mice exhibited memory deficits in Figure 1C. Overall, the above data emphasise the pivotal role of UTX in regulating cognitive performance.

### 3.2 UTX Neuronal cKO Leads to Hippocampal and Cortical Cell Death and Apoptosis

**FIGURE 2:**
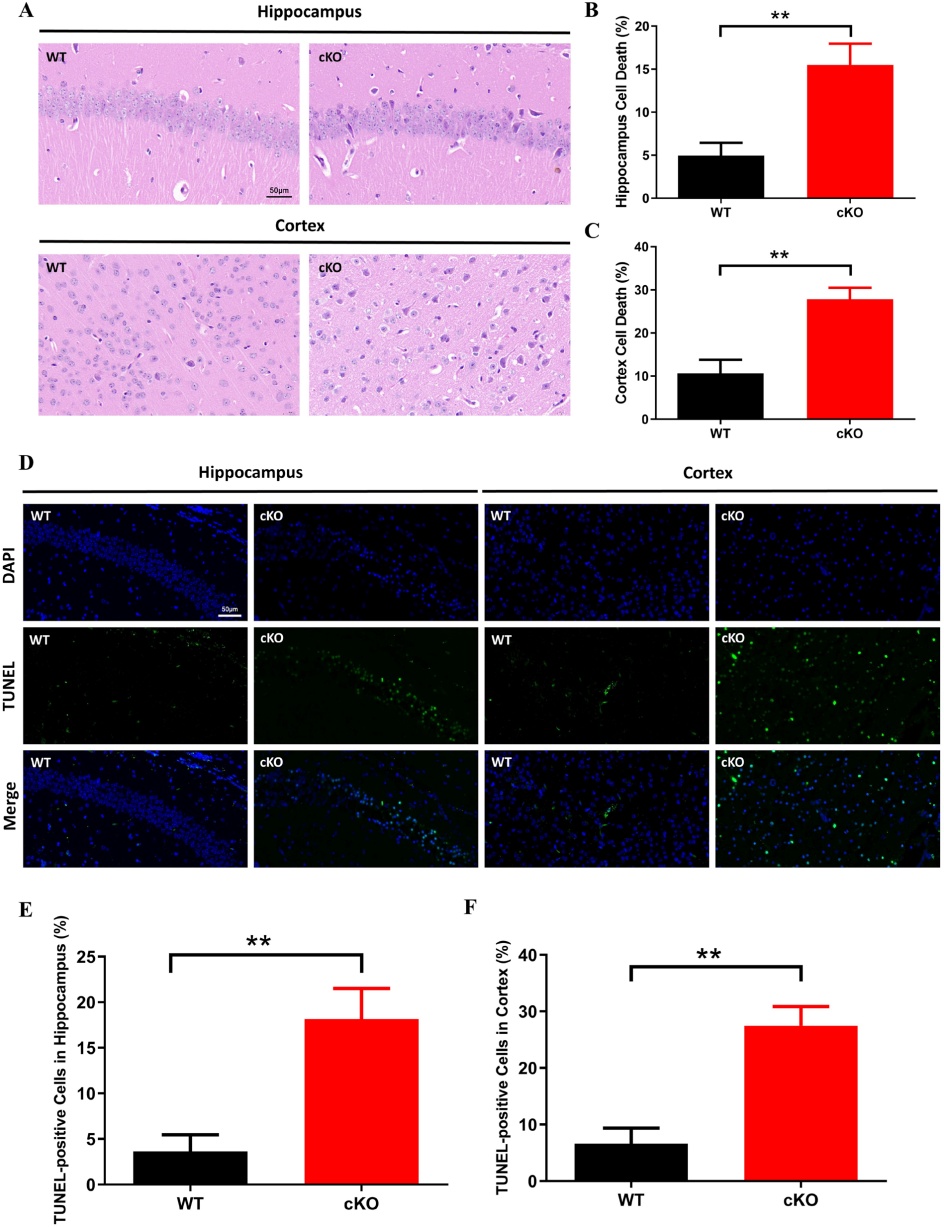
Effects of UTX on cell death and apoptosis in mouse hippocampus and cortex. (A) Representative images of H&E staining (magnification: 400×, scale bar: 50 μm). Quantitative analysis of dead cells in the hippocampus (B) and cortex (C). (D) Representative images of TUNEL staining (magnification: 400×, scale bar: 50 μm). TUNEL‐positive cells were stained green and nuclei were stained blue by DAPI. Quantitative analysis of TUNEL‐positive cells in the hippocampus (E) and cortex (F). Comparisons between the WT and *UTX* cKO groups were made by Student's *t*‐test. All data were expressed as mean ± SD (*n* = 3). **p < 0.01 versus WT group.

To further explore the adverse influences of *UTX* deficiency on the brain, H&E and TUNEL staining were used to observe the histopathological changes and apoptosis in the brain, respectively. H&E‐stained sections of the mouse hippocampus and cortex are shown in Figure 2A. There were no pathological morphological changes observed in the WT group, with closely arranged cells and clear nucleolus. In contrast, in the *UTX* cKO group, the damaged cells in the hippocampus and cortex appeared to have condensed nuclei, neuronal degeneration, and necrosis. Morphometric analysis indicated that the proportion of dead cells in the hippocampus (Figure 2B) and cortical area (Figure 2C) was significantly increased (*p* < 0.01) in the *UTX* cKO group compared with the WT group. Apoptosis is an important cellular event contributing to poor neurodevelopment, and as shown in the results, TUNEL staining was almost negative in the WT group, whereas the number of TUNEL‐positive cells in the hippocampus (Figure 2D) and cortex (Figure 2E) was obviously increased (*p* < 0.01) after *UTX* knockout, suggesting that *UTX* deletion was responsible for apoptosis in the hippocampus and cerebral cortex.

### 3.3 Metabolomic Profiling Between the WT and UTX cKO Groups

**FIGURE 3:**
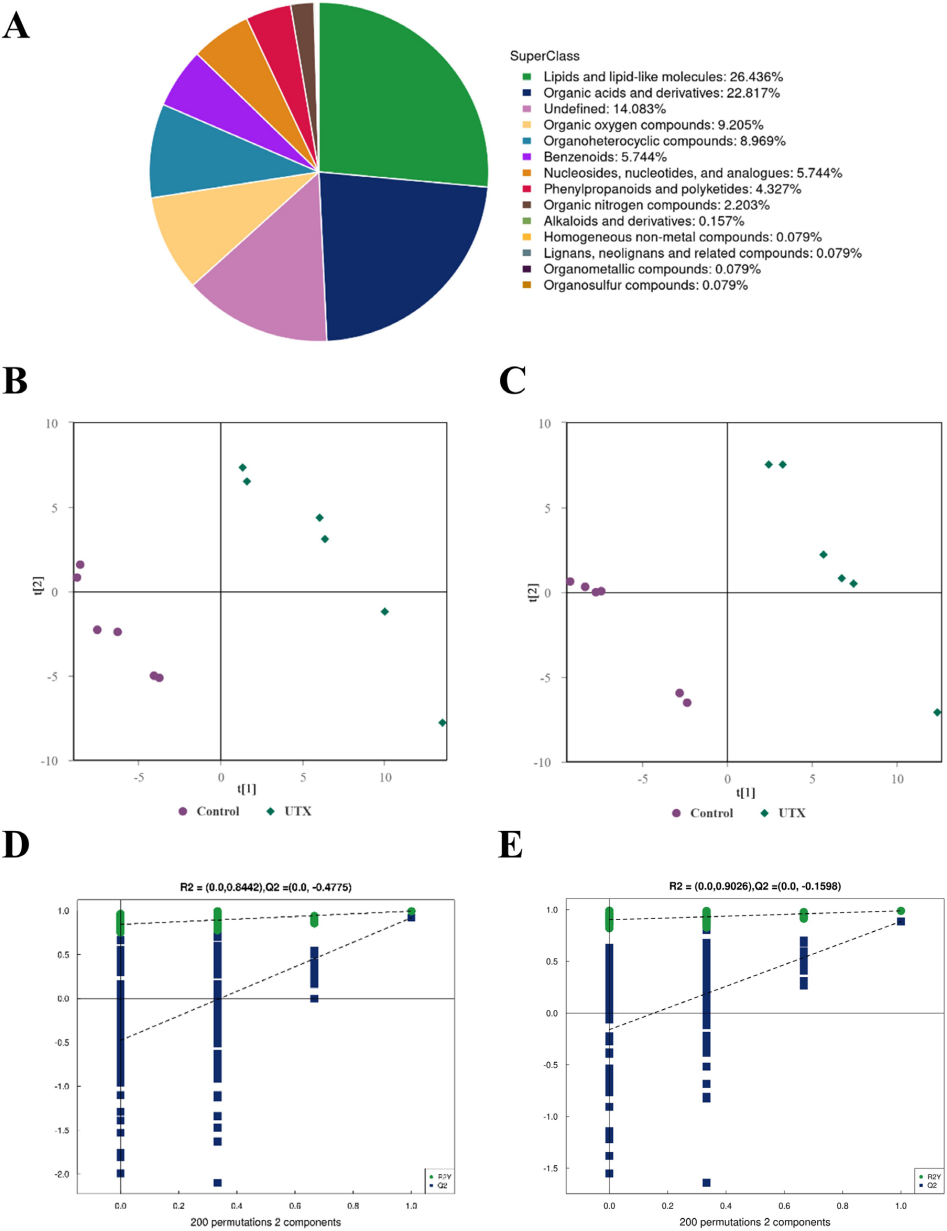
(A) Overview of identified metabolites. Score plots of the partial least squares discriminant analysis (PLS‐DA) model in the positive ion mode (B) and negative ion mode (C), the purple and green symbols represent brain tissue samples from the WT and *UTX* cKO groups, respectively. Permutation test (200 repeats) was used to validate the PLS‐DA model in the positive ion mode (D) and negative ion mode (E).

A total of 1271 metabolites were initially detected in brain tissues, mainly belonging to 14 different categories such as lipids and lipid‐like molecules, organic acids and derivatives, organic oxygen compounds, organoheterocyclic compounds, and benzenoids. However, 14.083% of the metabolites remained unidentified. The major metabolite categories and their percentages are displayed in Figure 3A. PLS‐DA was utilised to evaluate whether *UTX* deletion contributed to cerebral metabolic disturbances in subsequent experiments. In both positive and negative ion modes, the PLS‐DA score plots presented a good separation trend between the WT and *UTX* cKO groups, implying that *UTX* deletion resulted in obvious dissimilarity in metabolic profiles (Figure 3B,C). Furthermore, 200 times permutation tests were performed to ensure the reliability of the PLS‐DA model. As shown in Figure 3D,E, the *R* ^2^ and *Q* ^2^ values of the positive ion mode (*R* ^2^ = 0.8442, *Q* ^2^ = −0.4775) and the negative ion mode (*R* ^2^ = 0.9026, *Q* ^2^ = −0.1598) were acceptable, from which it can be concluded that the PLS‐DA model has good predictive ability without overfitting.

### 3.4 Identification of Differentially Expressed Metabolites

**FIGURE 4:**
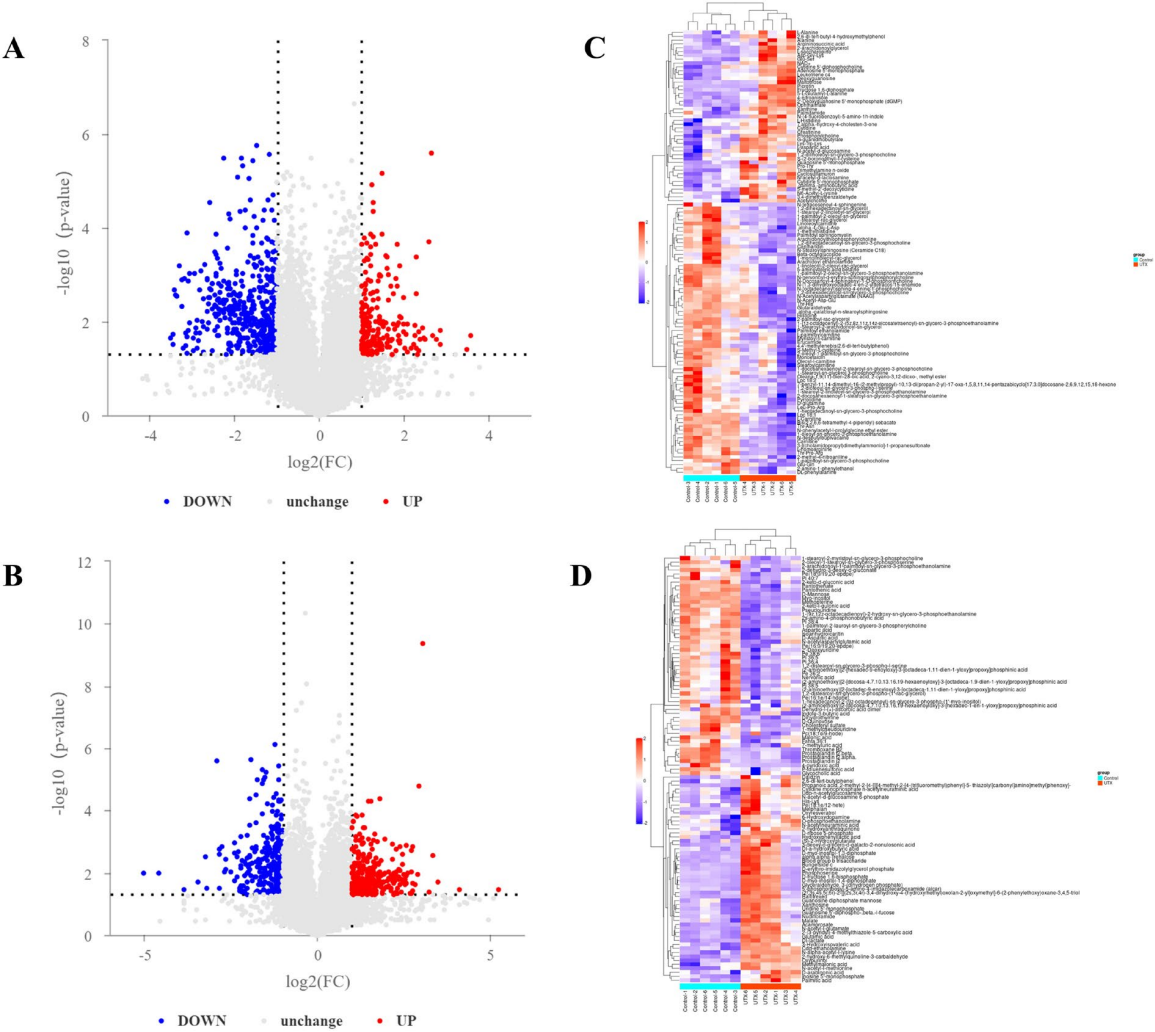
Volcano plot of metabolites in the WT and *UTX* cKO groups with FC > 1.5 or < 0.67 and *p* < 0.05. The red, grey, and blue dots in the figure represent metabolites that were upregulated, not significantly different, and downregulated in the *UTX* cKO group compared to the WT group in the positive ion mode (A) and negative ion mode (B), respectively. Heat map of differential metabolites detected between the WT (blue bars) and *UTX* cKO groups (red bars) in the positive (C) and negative ion modes (D), with red and purple representing upregulated and downregulated metabolites, respectively.

Based on fold changes (FC) > 1.5 or < 0.67 and *p* < 0.05, volcano plots were utilised to visualise the metabolites that were upregulated and downregulated in the *UTX* cKO group compared with the WT group. As a result, 103 metabolites were identified in the positive ion mode (Figure 4A), of which 39 were upregulated and 64 were downregulated, and 168 metabolites were identified in the negative ion mode, of which 43 were upregulated and 35 were downregulated (Figure 4B).

A total of 223 significant differential metabolites (116 in the positive ion mode and 107 in the negative ion mode) were eventually screened on the basis of VIP > 1.0 and *p* < 0.05. The heat map reflects the relative amount of each metabolite in different colours, with red representing high levels and purple representing low levels, to reveal the overall variation in differential metabolite levels between the different groups. As observed in Figure 4C,D, those differential metabolites were clearly distinguished between the WT and *UTX* cKO groups in either the positive or negative ion mode. The above results further strengthen that UTX may be a critical modulator for brain metabolism.

### 3.5 UTX Deletion May Cause Alterations in Metabolic Pathways

**FIGURE 5:**
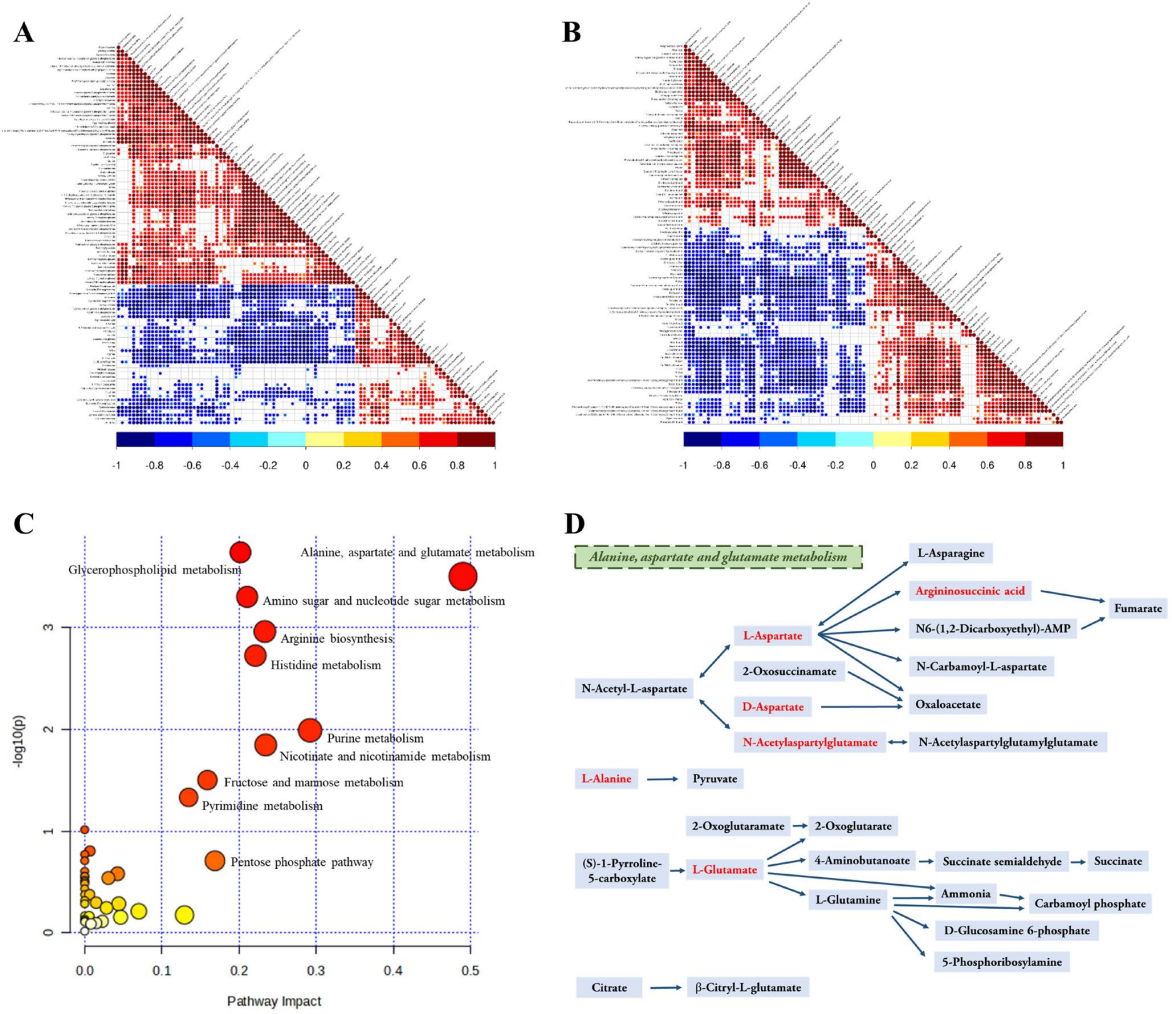
The correlation diagram of differential metabolites detected in the positive ion mode (A) and negative ion mode (B). Red, blue, and white mean positive, negative, and non‐significant correlations, respectively. Darker colours indicate a higher degree of positive or negative correlation. (C) KEGG pathway analysis of differential metabolites by MetaboAnalyst 6.0. The size and colour of the circle reflect the impact values (larger circles represent higher impact values) and the *p*‐values (redder circles represent lower *p*‐values and yellower circles represent higher *p*‐values), respectively. (D) Overview of the major metabolic pathway. Metabolites identified in the study were highlighted in red.

**FIGURE 6:**
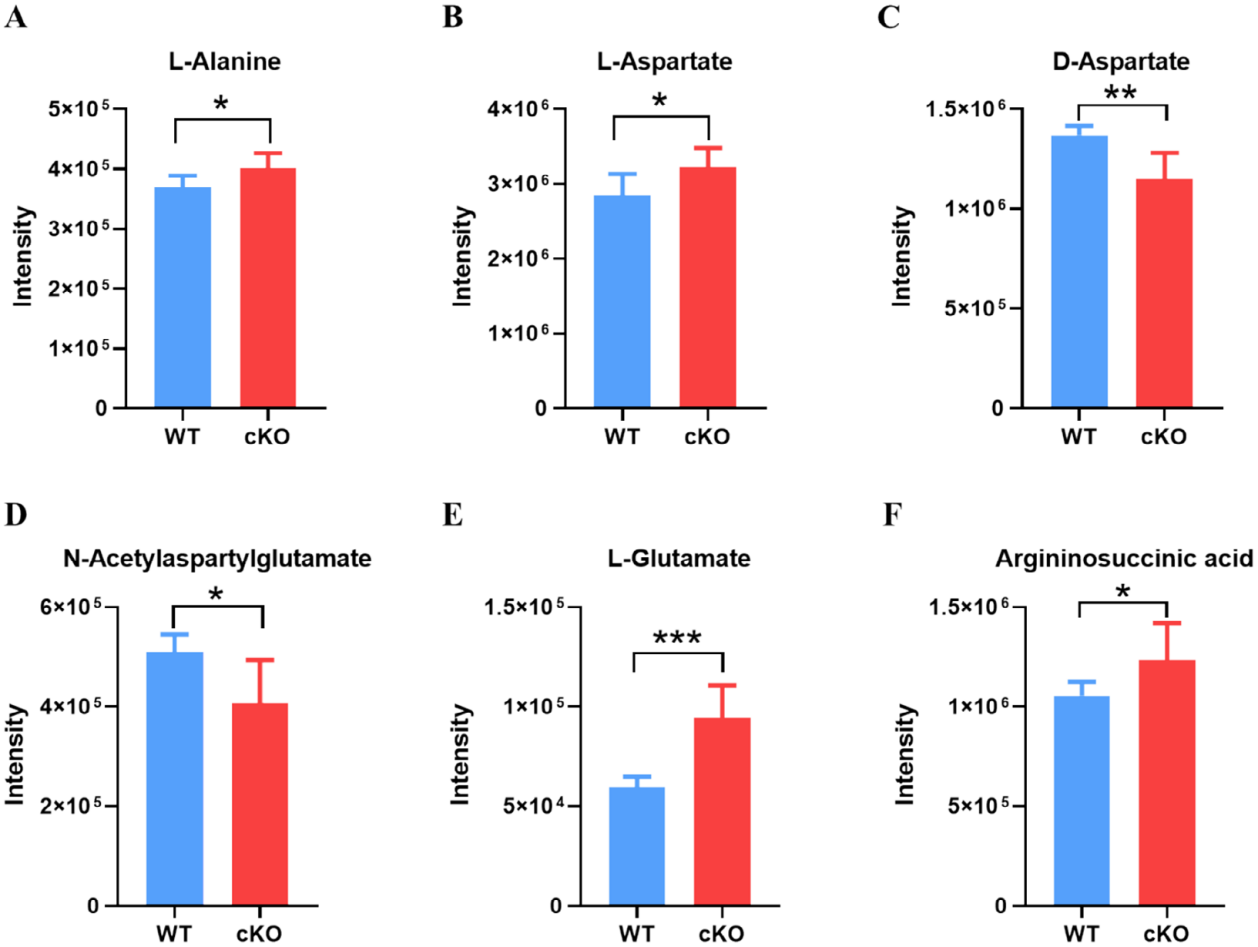
Main significantly affected metabolites in brain tissues between the WT and *UTX* cKO groups, including (A) L‐alanine, (B) L‐aspartate, (C) D‐aspartate, (D) N‐acetylaspartylglutamate, (E) L‐glutamate, and (F) argininosuccinic acid. Comparisons between the WT and *UTX* cKO groups were made using the Student's *t*‐test. The intensity of each metabolite was obtained by statistical analysis of its peak area. All data were expressed as mean ± SD (*n* = 6). *p < 0.05, **p < 0.01, ***p < 0.001 vs. WT group.

Pearson's correlation coefficient was used to analyse the correlation between differential metabolites in brain tissues, which helps to understand the inter‐regulatory relationship between different metabolites. As shown in Figure 5A,B, most of the metabolites are positively (red dots) or negatively (blue dots) correlated with each other in both the positive and negative ion modes, where positively correlated metabolites probably originate from the same metabolic pathways. Subsequently, to detect the critical pathway involved in the pathogenicity of *UTX* deficiency in the brain, 223 significant differential metabolites were imported into MetaboAnalyst software for pathway analysis. As seen in Figure 5C and Table 1, alanine, aspartate, and glutamate metabolism were determined to be the most influenced metabolic pathway. Figure 5D displays detailed information on this metabolic pathways, which covers the six differential metabolites identified in this investigation, including L‐alanine, L‐aspartate, D‐aspartate, N‐acetylaspartylglutamate, L‐glutamate, and argininosuccinic acid. Among them, the levels of L‐alanine, L‐aspartate, L‐glutamate, and argininosuccinic acid were significantly increased (*p* < 0.05, *p* < 0.05, *p* < 0.001, *p* < 0.05, Figure 6A,B,E,F) in the brains of *UTX* cKO mice compared to WT mice. Whereas the levels of D‐aspartate and N‐acetylaspartylglutamate in the brain were significantly decreased (*p* < 0.01, *p* < 0.05, Figure 6C,D) after neuronal *UTX* knockout.

**TABLE 1:** The KEGG pathway of differential metabolites in the brain of *UTX* cKO and WT mice.

No.	Metabolic pathways^a^	Metabolites^b^	*p*	Impact
1	Alanine, aspartate and glutamate metabolism	L‐alanine, L‐aspartate, D‐aspartate, N‐acetylaspartylglutamate, L‐glutamate, argininosuccinic acid	0.00	0.49
2	Purine metabolism	D‐ribose 5‐phosphate, deoxyguanosine, 2′‐deoxyguanosine 5′‐monophosphate, inosine 5′‐monophosphate, adenosine 5′‐monophosphate, xanthine, xanthosine	0.01	0.29
3	Nicotinate and nicotinamide metabolism	L‐aspartate, NAD+, N1‐methyl‐2‐pyridone‐5‐carboxamide	0.01	0.23
4	Arginine biosynthesis	L‐glutamate, N‐acetyl‐L‐glutamate, L‐aspartate, N‐(L‐arginino)succinate	0.00	0.23
5	Histidine metabolism	L‐histidine, 1‐methylhistidine, L‐aspartate, L‐glutamate	0.00	0.22
6	Amino sugar and nucleotide sugar metabolism	3‐deoxy‐d‐glycero‐d‐galacto‐2‐nonulosonic acid, N‐acetyl‐D‐glucosamine, D‐mannose, N‐acetyl‐D‐glucosamine 6‐phosphate, UDP‐N‐acetyl‐alpha‐D‐glucosamine, guanosine diphosphate mannose, CMP‐N‐acetylneuraminate	0.00	0.21
7	Glycerophospholipid metabolism	Ethanolamine phosphate, cytidine 5′‐diphosphocholine, phosphatidylethanolamine, choline phosphate, CDP‐choline, 1‐acyl‐sn‐glycero‐3‐phosphocholine, acetylcholine	0.00	0.20

^a^ Metabolic pathways with impact values greater than 0.2.

^b^ Metabolites involved in metabolic pathways identified in this study.

## 4 Discussion

The processes of neurodevelopment are precisely regulated by multiple epigenetic mechanisms [26]. Among them, several histone lysine demethylases (KDMs) have been identified as important players participating in neurodevelopment by modulating diverse cellular processes, such as chromatin structure and gene transcription [27]. Nowadays, the relationship between KDM6 family member UTX and neurodevelopment has attracted increasing attention. Considering that the role of UTX in the central nervous system (CNS) is yet to be determined, this study attempted to explore the impact of UTX on neurodevelopment in *UTX* cKO mice and further reveal the underlying mechanisms using untargeted metabolomics approaches.

Neurodevelopmental disorders have serious negative impacts on brain function, with cognitive deficits in particular regarded as a typical manifestation [28, 29]. Previous studies have preliminarily demonstrated that *UTX* deletion could induce impaired cognitive function in mice [30]. In our study, we first designed the MWM test to investigate whether *UTX* cKO mice exhibit cognitive dysfunction. Consistent with the previous study, our results showed that *UTX* cKO mice performed worse than WT mice in both the spatial learning trial and probe memory trial, reconfirming that *UTX* knockout may be associated with cognitive impairment. In subsequent pathology experiments, H&E staining and TUNEL staining were further employed to visualise the hippocampal and cortical cell damage in mice. As a result, more pronounced cell death and apoptosis were observed in the cortex and hippocampus of *UTX* cKO mice compared to WT mice. During neurodevelopment, it is known that apoptosis is a fundamental physiologic process that plays a prominent role in maintaining cellular homeostasis by selectively deleting redundant, faulty, and unused neurones [31, 32]. Of particular concern, increased apoptosis could trigger widespread neurodegeneration in the developing brain, which correlates with cognitive decline [33, 34]. Combining previous studies and our findings, we hypothesise that *UTX* deletion may be an adverse event that induces massive apoptosis, ultimately leading to neurodevelopmental abnormalities and cognitive deficits.

Next, to investigate the potential mechanisms underlying the effects of UTX on neurodevelopment, we performed brain metabolic profiling on WT and *UTX* cKO mice. The results of metabolomics analysis showed extensive metabolic changes in the brain between the two groups, and a total of 223 metabolites (116 in the positive ion mode and 107 in the negative ion mode) were eventually selected as the significant differential metabolites. More importantly, the following pathway analysis identified that alanine, aspartate, and glutamate metabolism may be the most important metabolic pathways. It is noteworthy that the six differential amino acids detected in brain tissues, including L‐alanine, L‐aspartate, D‐aspartate, N‐acetylaspartylglutamate and L‐glutamate, are all located in the alanine, aspartate, and glutamate metabolic pathway, and we hypothesised that UTX may affect neurological function by regulating these metabolites in brain tissue.

Among the metabolites identified above, the one that particularly caught our attention was L‐glutamate, which showed the most significant difference between the two groups (*p* < 0.001). It is well established that glutamate is not only the most abundant amino acid in the mammalian CNS but also a major excitatory neurotransmitter that plays a crucial role in higher brain functions such as learning and memory [35, 36, 37]. Accumulating evidence highlighted that glutamate participates in regulating and balancing excitatory and inhibitory inputs in the CNS to achieve stable neuronal activity and that once this balance is disturbed, it may lead to neurodevelopmental disorders and various neurological diseases [38]. Exposure to glutamate might be the reason for cell death, especially apoptosis in brain cells [39]. In addition, excess glutamate in the brain has been shown to be excitotoxic and potentially impair higher cognitive functions [40, 41]. Several studies pointed out that glutamate storm may activate excessive dendritic pruning and dendritic apoptosis during critical periods of neurodevelopment, which has been linked to cognitive impairment‐related diseases such as Alzheimer's disease and schizophrenia [42, 43]. Of particular note, our data indicated that L‐glutamate was significantly increased in the brain of *UTX* cKO mice compared to WT mice. Consistently, recent findings also support that *KDM6A* (*UTX*) plays a key role in glutamate metabolism [44]. In contrast to our findings, glutamate was reduced in anoikis‐resistant cancer cells in response to *KDM6A/B* inhibition [44]. We consider that the differential regulation of UTX in different cells and tissues, as well as the fact that the experimental conclusions were also influenced by the inhibitory effect of *KDM6B* (lysine‐specific demethylase 6B; KDM6B is a histone demethylase in the same family as UTX), may explain the discrepancy between these results and ours. From the foregoing, it can be speculated that *UTX* deletion may trigger cell apoptosis through increasing L‐glutamate content in the brain, thereby affecting neurodevelopment and eventually causing the emergence of cognitive deficits.

D‐aspartate is an atypical amino acid that is abundant in the developing mammalian brain [45]. There is growing evidence supporting that D‐aspartate possesses important regulatory properties in the nervous system and is involved in critical brain processes such as synaptic plasticity, brain development, and cognition [45, 46, 47]. In particular, D‐aspartate is known to be beneficial in improving cognition, and chronic administration of D‐aspartate is considered a treatment for cognitive impairment [48, 49]. In agreement with the above, other studies have noted that altered D‐aspartate metabolism in the brain may affect normal brain neurodevelopment and its associated phenotypes, as mice with D‐aspartate depletion exhibit recognition memory deficits [50]. In the present study, the level of D‐aspartate in the brain was significantly decreased after neuronal *UTX* knockout. We consider that this phenomenon may limit the benefits of D‐aspartate in the brain, correspondingly causing neurodevelopmental and cognitive dysfunction in *UTX* cKO mice.

Besides, N‐acetylaspartylglutamate was also identified as the main differential metabolite in this study, with significantly decreased levels in the brains of *UTX* cKO mice. On the one hand, N‐acetylaspartylglutamate can be hydrolysed to glutamate and N‐acetylaspartate by glutamate carboxypeptidase II (GCPII) in the nervous system [51]. Based on this information, we propose that *UTX* deletion may promote the hydrolysis of N‐acetylaspartylglutamate and result in lower levels of N‐acetylaspartylglutamate and higher levels of glutamate in the brain, which may adversely affect neurodevelopment. On the other hand, as the third most abundant neurotransmitter in the mammalian nervous system, N‐acetylaspartylglutamate has modulatory actions at various synaptic functions in different brain regions, which in turn mediates a pro‐cognitive effect [52, 53, 54]. Therefore, the cerebral N‐acetylaspartylglutamate alteration caused by *UTX* knockout may limit its beneficial effects in regulating cognitive functions and further exacerbate cognitive deficits in *UTX* cKO mice.

Finally, the levels of L‐alanine, L‐aspartate, and argininosuccinic acid were significantly increased in the *UTX* cKO group compared to the WT group. Among them, L‐alanine has been reported to significantly increase acetylcholinesterase content and (Na^+^, K^+^)‐ATPase activity, thereby promoting normal neural development [55]. Besides, most studies have proved that L‐aspartate is an important transmitter in various brain regions, while its function in the CNS remains controversial [56, 57]. The controversy over the effects of L‐aspartate may be similar to that of glutamate, as they both act on N‐methyl‐D‐aspartate (NMDA) receptors [56]. Under normal conditions, L‐aspartate may promote neurodevelopment by activating NMDA receptors, but accumulation of L‐aspartate may lead to apoptosis in the nervous system [56]. Additionally, the available data suggest that argininosuccinic acid is neurotoxic, as higher doses of argininosuccinic acid were found to be toxic to developing astrocytes and neurones, shedding light on how argininosuccinic acid may influence brain development [58]. Since the studies of the above three metabolites in neurodevelopment are rather limited, we tentatively suggest that *UTX* knockout may stimulate the brain to generate self‐protective responses by increasing L‐alanine levels, whereas increases in L‐aspartate and argininosuccinic acid may be involved in UTX‐induced neurodevelopmental damage.

Further, the specific pathways by which *UTX* regulates metabolites are also worth considering. In our experiments, neuronal *UTX* knockout ultimately led to a decrease in UTX protein levels, which inevitably induced metabolic disturbances in brain tissue. Since *UTX* is an important transcription factor that regulates gene transcription, UTX may have a regulatory effect on metabolic pathway regulators, and UTX knockdown may lead to changes in metabolites by affecting the expression of these regulators. In addition, previous studies have found that UTX has a regulatory effect on metabolic pathway regulators [30, 59, 60, 61]. Therefore, UTX knockdown may lead to changes in metabolites by affecting the expression of these regulators. All of the above may contribute to the metabolic disorders in brain tissue caused by UTX, but the specific regulatory mechanisms still need to be clarified by experiments such as target interventions.

Altogether, this study successfully excavated key metabolites that may be involved in the neurodevelopmental impairments caused by *UTX* deficiency, contributing to further elucidation of the pathological process of neurodevelopmental and cognitive disorders, as well as providing potential candidate targets for early intervention or prevention of neurodevelopmental diseases.

Nevertheless, there are some limitations in the current study. On the one hand, the biological sample size is relatively small in this investigation, and behavioural studies used to assess cognitive function in experimental animals have only been conducted with water maze experiments. Other behavioural tests, such as passive avoidance paradigms, can help to provide more evidence about cognitive function. On the other hand, untargeted metabolomics was employed in this study for preliminary exploration, and further targeted metabolomics, or even the combination of transcriptomics, proteomics, and other technologies, are needed to fully understand the mechanism of UTX involved in the regulation of neurodevelopment. In a word, larger samples, more behavioural experiments, and more analytical techniques are required to further validate the conclusions of this study.

## 5 Conclusion

Collectively, we conducted a brain metabolomics study to investigate the correlation between UTX and neurodevelopment through UPLC‐MS/MS‐based untargeted metabolomics. The results demonstrate that UTX deficiency could be a risk factor for neurodevelopment as it leads to brain cell death and apoptosis and cognitive decline. Simultaneously, our findings provide original evidence that the brain metabolic profiles of UTX cKO mice are apparently different from those of WT mice. Further pathway analysis reveals that alanine, aspartate, and glutamate metabolism may be the most important metabolic pathways and that altered metabolites involved in this pathway, especially D‐aspartate, N‐acetylaspartylglutamate, L‐glutamate, and argininosuccinic acid, may be potential mechanisms for the neurodevelopmental deficits induced by UTX deficiency. To the best of our knowledge, this is the first report to illuminate how UTX affects neurodevelopment from a metabolic perspective, and we anticipate that this study will facilitate exploration in this field.

## Author Contributions

**Lin Chen:** project administration (equal), writing – original draft (equal). **Maozhu Liu:** project administration (equal). **Xinhua Dai:** project administration (equal). **Cuilin He:** project administration (equal). **Kejing Wang:** project administration (equal). **Jinhua Tang:** project administration (equal). **Yang Yang:** conceptualization (equal), funding acquisition (supporting).

## Conflicts of Interest

The authors declare no conflicts of interest. Graphical abstract was created by Figdraw (www.figdraw.com).

## Data Availability

The raw data supporting the results of this study are available from the corresponding author.

## References

[1] M. J. Nona , C. Maureen , N. M. Shannon , and W. A. Petri, Jr. , “The Impact of Systemic Inflammation on Neurodevelopment,” Trends in Molecular Medicine 24, no. 9 (2018): 794–804, 10.1016/j.molmed.2018.06.008. 30006148 PMC6110951

[2] C. Gonçalves , E. Le Boiteux , P. Arnaud , and B. Costa , “HOX Gene Cluster (De)regulation in Brain: From Neurodevelopment to Malignant Glial Tumours,” Cellular and Molecular Life Sciences 77, no. 19 (2020): 3797–3821, 10.1007/s00018-020-03508-9. 32239260 PMC11105007

[3] P. Miguel , L. Pereira , P. Silveira , and M. Meaney , “Early Environmental Influences on the Development of children's Brain Structure and Function,” Developmental Medicine and Child Neurology 61, no. 10 (2019): 1127–1133, 10.1111/dmcn.14182. 30740660

[4] E. Bora , “A Neurodevelopment and Neuroplasticity‐Based Framework for Early Intervention in Psychotic Disorders,” Psychological Medicine 48, no. 3 (2017): 353–361, 10.1017/s0033291717002045. 28799518

[5] Y. Xiaoyang , X. Beisi , M. Brett , et al., “Differentiation of Human Pluripotent Stem Cells Into Neurons or Cortical Organoids Requires Transcriptional Co‐Regulation by UTX and 53BP1,” Nature Neuroscience 22, no. 3 (2019): 362–373, 10.1038/s41593-018-0328-5. 30718900 PMC6511450

[6] Q. Tang , S. Zhang , S. Dai , et al., “UTX Regulates Human Neural Differentiation and Dendritic Morphology by Resolving Bivalent Promoters,” Stem Cell Reports 15, no. 2 (2020): 439–453, 10.1016/j.stemcr.2020.06.015. 32679064 PMC7419705

[7] X. Lei and J. Jiao , “UTX Affects Neural Stem Cell Proliferation and Differentiation Through PTEN Signaling,” Stem Cell Reports 10, no. 4 (2018): 1193–1207, 10.1016/j.stemcr.2018.02.008. 29551674 PMC5998300

[8] Z. Jinzhi , T. Yingbing , Z. Huihui , et al., “p53 Mutant p53(N236S) Induces Neural Tube Defects in Female Embryos,” International Journal of Biological Sciences 15, no. 9 (2019): 2006–2015, 10.7150/ijbs.31451. 31523200 PMC6743294

[9] K. Mehrnoosh , J. Ehsan , B. Fatemeh , et al., “Kabuki Syndrome: Identification of Two Novel Variants in KMT2D and KDM6A,” Molecular Syndromology 12, no. 2 (2021): 118–126, 10.1159/000513199. 34012382 PMC8114050

[10] C. Yiheng , L. Tianyuan , P.‐K. Ulrika , et al., “Genomic Atlas of the Plasma Metabolome Prioritizes Metabolites Implicated in Human Diseases,” Nature Genetics 55, no. 1 (2023): 44–53, 10.1038/s41588-022-01270-1. 36635386 PMC7614162

[11] H. J. Caroline , I. Julijana , and S. Gary , “Metabolomics: Beyond Biomarkers and Towards Mechanisms,” Nature Reviews. Molecular Cell Biology 17, no. 7 (2016): 451–459, 10.1038/nrm.2016.25. PMC5729912 26979502

[12] D. Wishart , “Metabolomics for Investigating Physiological and Pathophysiological Processes,” Physiological Reviews 99, no. 4 (2019): 1819–1875, 10.1152/physrev.00035.2018. 31434538

[13] C. S.‐R. Alexandra , G. C. Simona , D. S. Stacy , and A. M. John , “Untargeted Metabolomics Strategies‐Challenges and Emerging Directions,” Journal of the American Society for Mass Spectrometry 27, no. 12 (2016): 1905, 10.1007/s13361-016-1469-y. PMC5110944 27624161

[14] S. Yaping and L. Weidong , “Recent Advances and Perspectives of Metabolomics‐Based Investigations in Parkinson's Disease,” Molecular Neurodegeneration 14, no. 1 (2019): 3, 10.1186/s13024-018-0304-2. 30634989 PMC6330496

[15] A. H. F. Tiago , P. V. R. Cristiana , A. Rúben , et al., “The Impact of the Serum Extraction Protocol on Metabolomic Profiling Using UPLC‐MS/MS and FTIR Spectroscopy,” ACS Omega 8, no. 23 (2023): 20755–20766, 10.1021/acsomega.3c01370. 37323376 PMC10237515

[16] A. Botas , H. Campbell , X. Han , and M. Maletic‐Savatic , “Metabolomics of Neurodegenerative Diseases,” International Review of Neurobiology 122 (2015): 53–80, 10.1016/bs.irn.2015.05.006. 26358890

[17] W. Fulin , L. Sihan , F. Dongxing , et al., “Neuroprotective Effects and Metabolomics Study of Protopanaxatriol (PPT) on Cerebral Ischemia/Reperfusion Injury In Vitro and In Vivo,” International Journal of Molecular Sciences 24, no. 2 (2023): 1789, 10.3390/ijms24021789. 36675303 PMC9861888

[18] G. Sangeetha and S. Uma , “Metabolomics of Neurological Disorders in India,” Analytical Science Advances 2 (2021): 594–610, 10.1002/ansa.202000169. 38715858 PMC10989583

[19] G. B. Moreau , R. Girija , L. C. Heather , et al., “Childhood Growth and Neurocognition Are Associated With Distinct Sets of Metabolites,” eBioMedicine 44 (2019): 597–606, 10.1016/j.ebiom.2019.05.043. 31133540 PMC6604877

[20] R. Beate , Y. Qi , U. Karan , et al., “Untargeted Metabolomics Screen of Mid‐Pregnancy Maternal Serum and Autism in Offspring,” Autism Research 13, no. 8 (2020): 1258–1269, 10.1002/aur.2311. 32496662 PMC13292088

[21] J. Gorski , T. Talley , M. Qiu , L. Puelles , J. Rubenstein , and K. Jones , “Cortical Excitatory Neurons and Glia, but Not GABAergic Neurons, Are Produced in the Emx1‐Expressing Lineage,” Journal of Neuroscience 22, no. 15 (2002): 6309–6314, 10.1523/jneurosci.22-15-06309.2002. 12151506 PMC6758181

[22] P. T. Kevin , T. M. Brian , L. B. Michelle , A. C. Sally , and L. B. Stephanie , “Ash1 l Loss‐of‐Function Results in Structural Birth Defects and Altered Cortical Development,” Brain. (2024): 218, Published ahead of print, 10.1093/brain/awae218.

[23] C. Vorhees and M. Williams , “Morris Water Maze: Procedures for Assessing Spatial and Related Forms of Learning and Memory,” Nature Protocols 1, no. 2 (2006): 848–858, 10.1038/nprot.2006.116. 17406317 PMC2895266

[24] Y. Yang , P. Xiang , Q. Chen , et al., “The Imbalance of PGD2‐DPs Pathway Is Involved in the Type 2 Diabetes Brain Injury by Regulating Autophagy,” International Journal of Biological Sciences 17, no. 14 (2021): 3993–4004, 10.7150/ijbs.60149. 34671214 PMC8495389

[25] E. Want , P. Masson , F. Michopoulos , et al., “Global Metabolic Profiling of Animal and Human Tissues via UPLC‐MS,” Nature Protocols 8, no. 1 (2013): 17–32, 10.1038/nprot.2012.135. 23222455

[26] R. D. Salinas , D. R. Connolly , and H. Song , “Invited Review: Epigenetics in Neurodevelopment,” Neuropathology and Applied Neurobiology 46, no. 1 (2020): 6–27, 10.1111/nan.12608. 32056273 PMC7174139

[27] H. Wang , B. Guo , and X. Guo , “Histone Demethylases in Neurodevelopment and Neurodegenerative Diseases,” International Journal of Neuroscience 134 (2023): 1372–1382, 10.1080/00207454.2023.2276656. 37902510

[28] F. D. Andrea and A. M. Melissa , “Neurodevelopmental Outcomes in Early Childhood,” Clinics in Perinatology 45, no. 3 (2018): 377–392, 10.1016/j.clp.2018.05.001. 30144844

[29] O. Can , M. B. Rachel , S. Sarah , and D. G. Kimberly , “Gene Transfer Therapy for Neurodevelopmental Disorders,” Developmental Neuroscience 43 (2021): 230–240, 10.1159/000515434. 33882495

[30] T. Gang‐Bin , Z. Yu‐Qiang , L. Pei‐Pei , et al., “The Histone H3K27 Demethylase UTX Regulates Synaptic Plasticity and Cognitive Behaviors in Mice,” Frontiers in Molecular Neuroscience 10 (2017): 267, 10.3389/fnmol.2017.00267. 28970783 PMC5609596

[31] S. Nemanja , H.‐T. Kazue , J.‐T. Vesna , and I. Nobuyuki , “Nonapoptotic Caspases in Neural Development and in Anesthesia‐Induced Neurotoxicity,” Trends in Neurosciences 45, no. 6 (2022): 446–458, 10.1016/j.tins.2022.03.007. 35491256 PMC9117442

[32] E. C. Catherine , “From Drug‐Induced Developmental Neuroapoptosis to Pediatric Anesthetic Neurotoxicity—Where Are We Now?,” Brain Sciences 6, no. 3 (2016): 32, 10.3390/brainsci6030032. 27537919 PMC5039461

[33] I. Chrysanthy , “Triggers of Apoptosis in the Immature Brain,” Brain & Development 31, no. 7 (2009): 488–492, 10.1016/j.braindev.2009.02.006. 19307071

[34] S. Kumari , R. Dhapola , and D. Reddy , “Apoptosis in Alzheimer's Disease: Insight Into the Signaling Pathways and Therapeutic Avenues,” Apoptosis: An International Journal on Programmed Cell Death 28 (2023): 943–957, 10.1007/s10495-023-01848-y. 37186274

[35] F. Fonnum , “Glutamate: a neurotransmitter in mammalian brain,” Journal of Neurochemistry 42, no. 1 (1984): 1–11, 10.1111/j.1471-4159.1984.tb09689.x. 6139418

[36] Y. Sun , T. Nguyen , A. Anderson , et al., “In Vivo Glutamate Sensing Inside the Mouse Brain With Perovskite Nickelate‐Nafion Heterostructures,” ACS Applied Materials & Interfaces 12, no. 22 (2020): 24564–24574, 10.1021/acsami.0c02826. 32383375

[37] C. Garin , N. Nadkarni , J. Pépin , J. Flament , and M. Dhenain , “Whole Brain Mapping of Glutamate Distribution in Adult and Old Primates at 11.7T,” NeuroImage 251 (2022): 118984, 10.1016/j.neuroimage.2022.118984. 35149230

[38] S. Sears and S. Hewett , “Influence of Glutamate and GABA Transport on Brain Excitatory/Inhibitory Balance,” Experimental Biology and Medicine 246, no. 9 (2021): 1069–1083, 10.1177/1535370221989263. 33554649 PMC8113735

[39] M. Ankarcrona , “Glutamate Induced Cell Death: Apoptosis or Necrosis?,” Progress in Brain Research 116 (1998): 265–272, 10.1016/s0079-6123(08)60442-2. 9932382

[40] B. Gruenbaum , A. Zlotnik , I. Fleidervish , A. Frenkel , and M. Boyko , “Glutamate Neurotoxicity and Destruction of the Blood‐Brain Barrier: Key Pathways for the Development of Neuropsychiatric Consequences of TBI and Their Potential Treatment Strategies,” International Journal of Molecular Sciences 23, no. 17 (2022): 9628, 10.3390/ijms23179628. 36077024 PMC9456007

[41] Y. Zhang , J. Chu , and G. Wong , “Cerebral Glutamate Regulation and Receptor Changes in Perioperative Neuroinflammation and Cognitive Dysfunction,” Biomolecules 12, no. 4 (2022): 597, 10.3390/biom12040597. 35454185 PMC9029551

[42] E. Parellada and P. Gassó , “Glutamate and Microglia Activation as a Driver of Dendritic Apoptosis: A Core Pathophysiological Mechanism to Understand Schizophrenia,” Translational Psychiatry 11, no. 1 (2021): 271, 10.1038/s41398-021-01385-9. 33958577 PMC8102516

[43] X. Liu , J. Yan , F. Liu , et al., “Overexpression of REST Causes Neuronal Injury and Decreases Cofilin Phosphorylation in Mice,” Journal of Alzheimer's Disease 87, no. 2 (2022): 873–886, 10.3233/jad-210285. 35404272

[44] A. A. Mohamed , R. S. M. Mohammed , M. H. Anwar , S. A. Turki , A. A. Nabil , and I. K. Mohammad , “Extracellular Matrix Detached Cancer Cells Resist Oxidative Stress by Increasing Histone Demethylase KDM6 Activity,” Saudi Journal of Biological Sciences 31, no. 1 (2023): 103871, 10.1016/j.sjbs.2023.103871. 38107766 PMC10724685

[45] F. Errico , R. Nisticò , A. Di Giorgio , et al., “Free D‐Aspartate Regulates Neuronal Dendritic Morphology, Synaptic Plasticity, Gray Matter Volume and Brain Activity in Mammals,” Translational Psychiatry 4, no. 7 (2014): e417, 10.1038/tp.2014.59. 25072322 PMC4119226

[46] G. Zachar , T. Jakó , I. Vincze , et al., “Age‐Related and Function‐Dependent Regional Alterations of Free L‐ and D‐Aspartate in Postembryonic Chick Brain,” Acta Biologica Hungarica 69, no. 1 (2018): 1–15, 10.1556/018.68.2018.1.1. 29575913

[47] G. Genchi , “An Overview on D‐Amino Acids,” Amino Acids 49, no. 9 (2017): 1521–1533, 10.1007/s00726-017-2459-5. 28681245

[48] V. de Rosa , A. Secondo , A. Pannaccione , et al., “D‐Aspartate Treatment Attenuates Myelin Damage and Stimulates Myelin Repair,” EMBO Molecular Medicine 11, no. 1 (2018): e9278, 10.15252/emmm.201809278. PMC6328990 30559305

[49] P. Enza , L. Livio , G. Francesca , et al., “D‐Aspartate Drinking Solution Alleviates Pain and Cognitive Impairment in Neuropathic Mice,” Amino Acids 48, no. 7 (2016): 1553–1567, 10.1007/s00726-016-2205-4. 27115160

[50] L. Barbara , P. Marco , D. R. Arianna , et al., “D‐Aspartate Oxidase Gene Duplication Induces Social Recognition Memory Deficit in Mice and Intellectual Disabilities in Humans,” Translational Psychiatry 12, no. 1 (2022): 305, 10.1038/s41398-022-02088-5. 35915065 PMC9343392

[51] Y. Gao , S. Xu , Z. Cui , et al., “Mice Lacking Glutamate Carboxypeptidase II Develop Normally, but Are Less Susceptible to Traumatic Brain Injury,” Journal of Neurochemistry 134, no. 2 (2015): 340–353, 10.1111/jnc.13123. 25872793

[52] J. Neale and T. Yamamoto , “N‐Acetylaspartylglutamate (NAAG) and Glutamate Carboxypeptidase II: An Abundant Peptide Neurotransmitter‐Enzyme System With Multiple Clinical Applications,” Progress in Neurobiology 184 (2020): 101722, 10.1016/j.pneurobio.2019.101722. 31730793

[53] J. Neale and R. Olszewski , “A Role for N‐Acetylaspartylglutamate (NAAG) and mGluR3 in Cognition,” Neurobiology of Learning and Memory 158 (2019): 9–13, 10.1016/j.nlm.2019.01.006. 30630041 PMC6397785

[54] C. Jiménez‐Espinoza , F. Marcano Serrano , and J. González‐Mora , “N‐Acetylaspartyl‐Glutamate Metabolism in the Cingulated Cortices as a Biomarker of the Etiology in ASD: A H‐MRS Model,” Molecules 26, no. 3 (2021): 675, 10.3390/molecules26030675. 33525414 PMC7866086

[55] C. Liapi , I. Feskou , A. Zarros , H. Carageorgiou , P. Galanopoulou , and S. Tsakiris , “Equilibrated Diet Restores the Effects of Early Age Choline‐Deficient Feeding on Rat Brain Antioxidant Status and Enzyme Activities: The Role of Homocysteine, L‐Phenylalanine and L‐Alanine,” Metabolic Brain Disease 23, no. 3 (2008): 289–301, 10.1007/s11011-008-9097-2. 18642068

[56] D. Balázs , A. Csillag , and G. Gerber , “L‐Aspartate Effects on Single Neurons and Interactions With Glutamate in Striatal Slice Preparation From Chicken Brain,” Brain Research 1474 (2012): 1–7, 10.1016/j.brainres.2012.07.049. 22871268

[57] A. Cavallero , A. Marte , and E. Fedele , “L‐Aspartate as an Amino Acid Neurotransmitter: Mechanisms of the Depolarization‐Induced Release From Cerebrocortical Synaptosomes,” Journal of Neurochemistry 110, no. 3 (2009): 924–934, 10.1111/j.1471-4159.2009.06187.x. 19549007

[58] C. Diez‐Fernandez , D. Hertig , M. Loup , et al., “Argininosuccinate Neurotoxicity and Prevention by Creatine in Argininosuccinate Lyase Deficiency: An In Vitro Study in Rat Three‐Dimensional Organotypic Brain Cell Cultures,” Journal of Inherited Metabolic Disease 42, no. 6 (2019): 1077–1087, 10.1002/jimd.12090. 30907007

[59] G. Lei , L. Zhuang , and B. Gan , “Targeting Ferroptosis as a Vulnerability in Cancer,” Nature Reviews. Cancer 22, no. 7 (2022): 381–396, 10.1038/s41568-022-00459-0. 35338310 PMC10243716

[60] R. Massart , J. P. Guilloux , V. Mignon , P. Sokoloff , and J. Diaz , “Striatal GPR88 Expression Is Confined to the Whole Projection Neuron Population and Is Regulated by Dopaminergic and Glutamatergic Afferents,” European Journal of Neuroscience 30, no. 3 (2009): 397–414, 10.1111/j.1460-9568.2009.06842.x. 19656174

[61] T. Laboute , J. Gandía , L. P. Pellissier , et al., “The Orphan Receptor GPR88 Blunts the Signaling of Opioid Receptors and Multiple Striatal GPCRs,” eLife 9 (2020): 9, 10.7554/eLife.50519. PMC7012601 32003745

